# Lack of Awareness, Body Confidence and Connotations of Sex: An Interpretative Phenomenological Analysis of Barriers Affecting the Decision to Attend Initial Cervical Cancer Screening

**DOI:** 10.1007/s11414-022-09819-y

**Published:** 2022-10-07

**Authors:** Phoebe Brook-Rowland, Katherine A. Finlay

**Affiliations:** 1 Institute of Sport and Physical Activity Research, University of Bedfordshire, Bedford, UK; 2 School of Psychology, University of Buckingham, Buckingham, UK; 3 School of Psychology and Clinical Language Sciences, University of Reading, Whiteknights, Reading, RG6 6AL UK

**Keywords:** Cervical cancer screening, Sex, Body confidence, Social comparison, Body disturbance

## Abstract

This study sought to understand how cervical cancer screening (CCS) awareness, sexual connotations and body image influenced the likelihood of CCS uptake in women yet to attend. Eleven females, aged 23–24, yet to attend CCS, were purposefully sampled. Interview transcripts were analysed using interpretative phenomenological analysis, generating three superordinate themes: (1) building screening expectations, (2) confronting sexual connotations and (3) growing pains. Findings demonstrated how a lack of awareness of CCS and the sexual connotations implicit in CCS acted as a barrier to attendance, exacerbated by negative body image comparisons between oneself and online or social media-based images. The perceived sexual connotations of CCS, and the resulting embarrassment, bolsters the case for self-screening, removing the need to attend clinic screening appointments. Reconceptualising screening using a theoretical model of the relationship between body image disturbances and body-focused screening behaviours among women, could lead to the development of pro-screening social media interventions.

## Introduction

Cervical cancer screening (CCS) is a preventative procedure to detect abnormalities in the cervix which could become cancerous.^1^ In England, individuals assigned female at birth are invited to CCS at age 25; however, a third do not attend their first screening.^2^ Frequently, practical issues such as location and time of CCS are cited as reasons for not engaging with this protective health behaviour.^3–5^ However, psychological factors are reported to be more predictive of screening status,^6^ such as fear, embarrassment, anxiety and stigma.^7–10^ Understanding what underpins psychological factors could facilitate more effective CCS attendance interventions.

However, what underpins the anxiety, fear and embarrassment has been minimally investigated. Body confidence influences initial pelvic examination attendance, including CCS.^11–13^ Social media use has been linked with decreased body satisfaction through comparisons.^14,15^ Similarly, pornography has been cited for increased concerns over vaginal appearance,^16^ reflected in blog posts questioning appropriate pubic hair styling for CCS.^17^ This indicates that women^1^ growing up in a digital age may have increased feelings of psychological discomfort over their own bodies, which could influence likelihood of CCS attendance.

**Figure 1:**
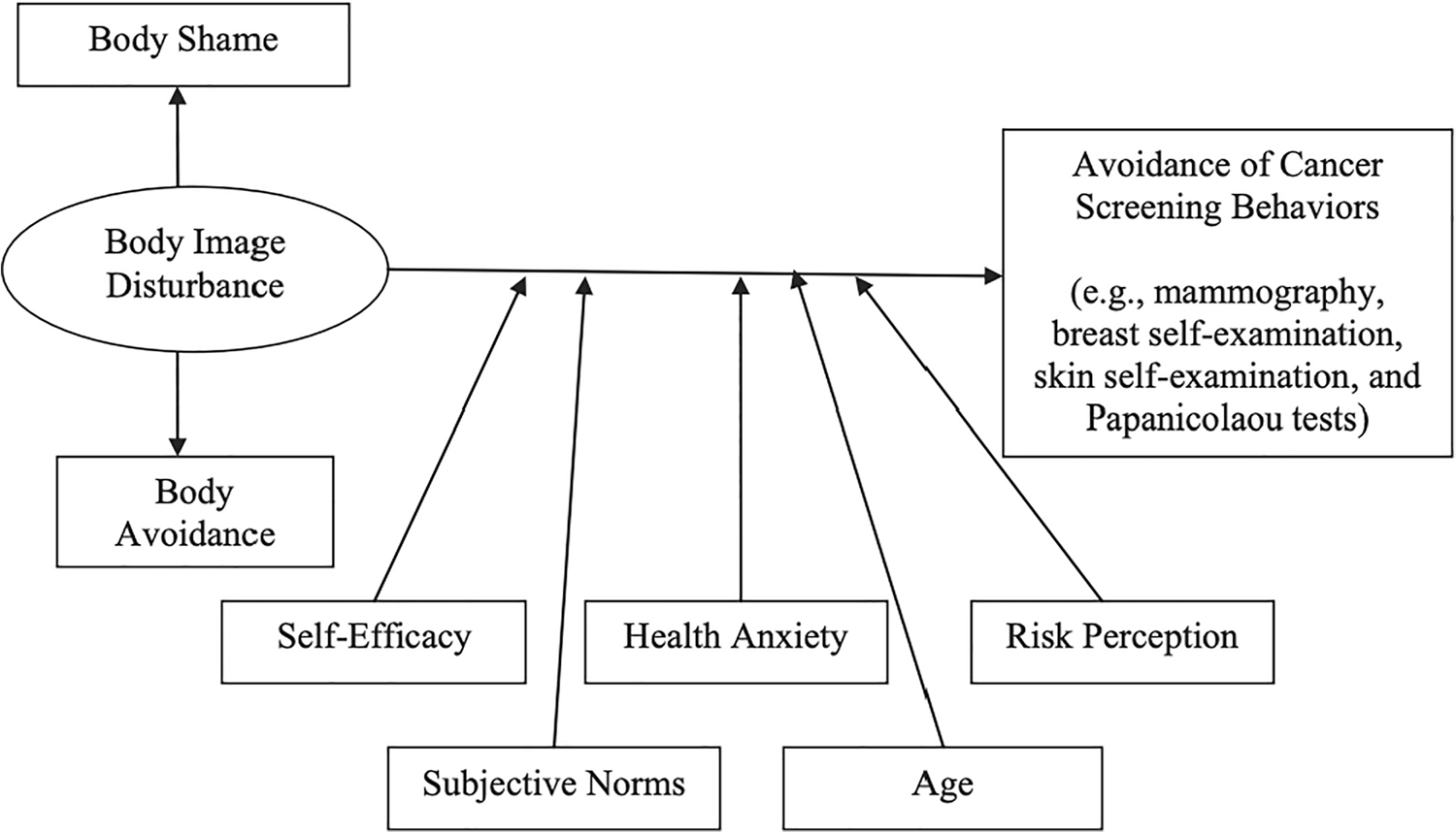
Ridolfi and Crowther’s (2013) model of relationship between body image disturbance and body-focused cancer screening among women

Ridolfi and Crowther’s (2013) model (see Fig. 1) theorises a relationship between body image disturbance and avoidance of cancer screening behaviours among women. Body image disturbance refers to persistent distress, dissatisfaction and dysfunction in relation to physical appearance^18^ and exists on a continuum from people living with psychiatric conditions such as body dysmorphia, through to ‘everyday’ concerns about one’s appearance.^19^ It manifests in body shame, the belief that one's body is inferior when compared with cultural beauty standards,^20^ and body avoidance actions, which are designed to limit the need to confront one's body image.^21^ The model suggests body image disturbance influences attendance at body-focused cancer screening but has not been applied or extended to CCS. It is likely that body image disturbance could be exacerbated in CCS due to the (necessary) focus on the sex organ.

It has been reported that the connotations of sex associated with CCS should be considered a hidden issue for screening uptake: inferred sexual connotations increase CCS anxieties in women 25–34.^22^ Additionally, the penetrative nature of the procedure causes psychological discomfort,^23^ with such sexual connections reflected in discussions online regarding which underwear is ‘too sexy’ for CCS.^17^ The doctor’s gender may mediate these concerns, as women often prefer female physicians for CCS.^3,5,24^ In women currently enrolled in screening programmes, it is evident in the literature that CCS can be conceptualised as having sexual overtones, but as yet research has not considered the impact these connotations have on women who are at the decision-making tipping point, shortly before their first screening invitation is received. It is possible that the strength of these connotations has increased due to the growth in social media use, and that this presents a major risk factor for people approaching their first invitation for CCS.

In addition, the salience of the sexual connotations may have been further increased due to the strength of the cognitive/societal link between screening and the human papillomavirus (HPV). In 2008, the UK began vaccinating girls between 11 and 13 to protect against HPV.^10^ HPV, a sexually transmitted infection (STI), is the cause of 99.7% of cervical cancer cases.^25,26^ In the UK, the cohorts of women who received their invitations to CCS from 2020 onwards were therefore the first cohort(s) to receive the HPV vaccine. For this group, receiving the HPV vaccine and facing the first invitation to CCS may increase the cognitive salience of the link between sex and screening. Indeed, the stigma attached to having an STI is an additional barrier for CCS attendance ^27^ and causes some women to delay disclosing a positive result.^28^ To increase the likelihood of attendance when invited, it is important to understand if women approaching their first CCS have specific concerns prior to attendance, and to identify the factors affecting their desire to attend. The voices of women yet to attend CCS are missing in the literature and exploratory studies could inform interventions targeting the initial decision-making stage, which is linked with subsequent CCS uptake. ^4^

Qualitative methods are recommended for exploratory studies ^29^ and have been used with multiple populations regarding CCS uptake. ^5,8,22,23^ Typically, qualitative CCS research focuses on specific sub-populations of particular interest in terms of screening uptake, such as older women,^5^ individual ethnic groups,^8^ women in homosexual relationships^23^ or women who are deliberately electing not to participate in screening.^22^ To date, no research has yet explored the viewpoints of women shortly before first screening; instead, it has considered only their perspectives *after* screening has commenced.^5,8,22,23^ Though it is recognised that there are likely to be commonalities between the themes evident in pre-existing literature, for example lack of knowledge about CCS, embarrassment and anxiety, there is an urgent need to consider novel perspectives which may impact upon the *initial decision* to attend screening. Such research could assess how an individual’s life experiences, perspectives and standpoints may affect their CCS decision-making processes.

To be able to fully reflect the voices of participants, interpretative phenomenological analysis (IPA) offers a qualitative method which attempts to reflect the lived experiences of participants.^30^ IPA has been noted for its utility in health psychology as it gives individuals a voice, allowing their lived experience to be heard with rich contextual detail.^31^ The current study used IPA to engage with the perspectives and experiences of women due to be invited to CCS in the next 2 years (participants currently aged 23–24) and who were the first cohorts to be offered the HPV vaccine during their schooling. This study aimed to explore the lived experience of considering future attendance at CCS screening, reflecting on the role of sex and stigma in that decision process.

## Methods

### Design

An exploratory qualitative design using semi-structured interviews and IPA was used.^32^ IPA is deemed particularly valuable in health psychology as it allows participants’ voices to heard via rich, multifaceted contextual details of their lived experience^31^ and has been used in studies on cancer, screening behaviours, body image and sexuality.^33–36^ Interpretative phenomenological analysis is a distinctive approach: it prioritises the detailed exploration of participants’ own reflections on their lived experiences and encourages depth of self-reflection by attempting to access and make sense of a participants' personal world, rather than delineating pre-existing theoretical perspectives.^32^ Individual, semi-structured interviews are considered a primary, effective method of accessing the depth of lived experience and the participant’s meaning-making processes, such as are required for effective phenomenological interpretation.^32^ One-to-one interviews were used to allow the participants to raise issues important to them and allow for further probing of their individual lived experience.^37^

### Participants

Purposive sampling of eleven participants ensured the inclusion criteria were met^11^: being female aged 23–24, and not yet attended CCS (full demographic details are provided in Table 1). Approximately 6–8 participants are recommended for thorough IPA if adequate depth of analysis and interview is to be retained,^32,37^ indicating that the current study met sampling adequacy. Aligning with previous studies on screening behaviours, broad demographic details were taken^3^; ethnicity, as women from ethnic minorities have been reported to be a third less likely to have attended cervical cancer screening than white British females^8^; sexual orientation, as women who are not heterosexual report differing health care concerns regarding cervical cancer screening uptake^38^; and educational attainment, as higher education attainment has been shown to correlate with increased likelihood of cervical cancer screening uptake and it has been suggested designing interventions without factoring educational attainment may limit their success.^39^ Whether participants had previously had the HPV vaccination or a pelvic examination was also recorded.

**Table 1:** Participant demographics

Pseudonym	Age	Sexual orientation	Education level	Ethnicity	HPV vaccine	Pelvic examination
Amy	24	Heterosexual	A levels	White British	Yes	Yes
Blaire	23	Heterosexual	Masters	Other mixed	Yes	Yes
Camilla	23	Bisexual	Diploma	Latina	Yes	No
Hayley	23	Heterosexual	Bachelor’s degree	White British	No	Yes
Jo	23	Heterosexual	Bachelor’s degree	British	No	Yes
Katie	23	Heterosexual	A levels	White British	No	No
Lori	23	Heterosexual	Masters	White British	Yes	No
Millie	24	Bisexual	A levels	British	Yes	Yes
Polly	23	Bisexual	Bachelor’s degree	British	Yes	Yes
Sarah	23	Heterosexual	A levels	White British	No	No
Vanessa	23	Heterosexual	Foundation degree	British	Yes	No

### Materials

A 9-item semi-structured interview schedule (Table 2) was designed in close consultation with the research literature to explore the following: participants’ pre-existing knowledge of CCS; their body confidence; the physicians’ gender; the sexual connotations of CCS; the impact of these factors on CCS uptake. The prioritisation of these topics within the interviews was informed by in-depth literature scoping of key research in the area.^3,11,22^

**Table 2:** Interview schedule

1	Can you tell me what you know about smear tests?
2	How do you personally feel about smear tests?
3	What do you think is influencing your thoughts about smear tests?
4	How do you think your body confidence and body image affects your likelihood of going to your first smear test?
5	What do you think has helped inform your beliefs about your body?
6	When you think about going for a smear test, how do you balance your ideas about both the medical view and sexual perception of your body?
7	How do you think your likelihood of attendance might be influenced by your sexual experiences?
8	To what extent do you think the gender of person doing the smear would influence your experience of a future smear test?
9	What do you personally think could be done to increase the likelihood of you attending your first smear test?

### Procedure

Interviews were conducted and transcribed verbatim by the research team. Eight interviews were in person and four via zoom, due to COVID-19 restrictions. Before the interview, participants were emailed a link to JISC online surveys to provide answers to the demographics questionnaire and give consent. The interview schedule was followed flexibly, allowing the participant to expand on areas of particular personal salience: the semi-structured nature of the interview allowed participants to raise issues important to their lived experience, which the researcher probed, enabling participant-led discussion.^32^ Interviews were recorded on an encrypted audio recording device and lasted between 43 and 94 min.

### Ethical Considerations

Ethical approval for the study was given by the University of Buckingham School of Psychology and Wellbeing ethics committee. Participants gave informed consent for use of their direct quotes and to be audio recorded. Participants were free to stop the recording and had the right to withdraw. Pseudonyms have been used and any identifiable information was removed at transcription to maintain anonymity and confidentiality. After the interview, details were provided for cervical cancer charities and information portals for further research into CCS.

### Analytic Steps for IPA

IPA was used. Through a process of listening and reading, the researchers familiarised themselves with the interviews individually, ensuring each participant’s voice was heard. Identification of descriptive comments, linguistic observation and conceptual meanings led to formation of exploratory themes. Their combination generated themes firmly rooted in the data. Cross-case analysis commenced after all interviews were ideographically analysed. Emergent themes were clustered to create subordinate themes; groupings of subordinate themes then determined superordinate themes. The researcher kept a reflexive log to facilitate bracketing-off of assumptions, maintaining particular awareness of any assumptions which held heightened salience as a result of the authors’ academic research/practice work in health psychology and specialisms in physical health and in managing chronic, long-term health conditions.^32^ To retain fidelity and maintain an idiographic stance, triangulation of super and subordinate themes was completed with the second author who has significant experience in IPA. Discrepancies were resolved in close consultation with the data and the researcher’s reflexive log. Aligned with the double hermeneutic circle, the researcher recognises their interpretation is informed by their contextual background and therefore may not be the only interpretation.^40^

## Results

Analysis identified three superordinate themes: (1) building screening expectations, (2) confronting sexual connotations and (3) growing pains. Analysis demonstrated the challenges inherent in the process of gaining an understanding of a procedure which participants were yet to have, the implications of the procedure due to the involvement of a sex organ, and a progressive movement away from a pervasive social comparison culture towards a more self-focused position in which CCS could be accepted. Superordinate themes and subordinate themes are presented in Table 3 and their prevalence among participants in Table 4.

**Table 3:** Superordinate themes and subordinate themes

Superordinate themes	Subordinate themes
Building screening expectations	Elusive information
	Anticipating a judgemental exchange
	Solace in one of many
Confronting sexual connotations	The salience of sex
	Projecting porn-like standards
	Comforted by female familiarity
Growing pains	Contending with comparison culture
	Becoming the focus

**Table 4:**
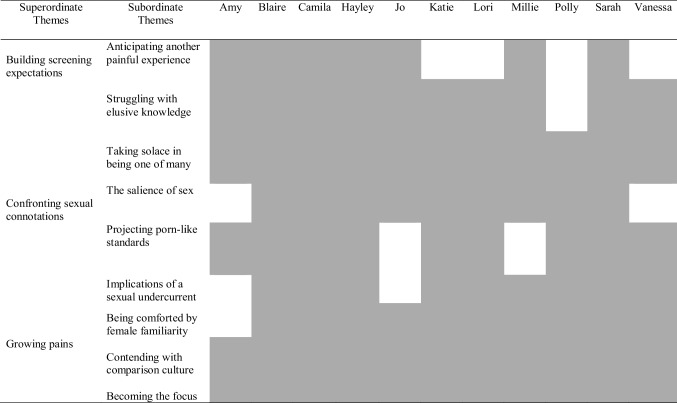
Prevalence of themes by participant

Greyed out boxes indicate which participants are represented within each theme

### Building Screening Expectations

The first superordinate theme considers the challenge of conceptualising a procedure yet to be had. Although participants often felt they had inadequate information, some drew on analogous experiences of pelvic examinations or found safety in the experience of others.

## Elusive Information

Nearly all participants noted how little information on CCS they had. However, they attributed this differently:

You never know until you go through it, so you have to just do it. I don’t know very many people personally who talk about this, you can’t just walk into the office and be like ‘so had my smear test this week, how did it go? Oh, let’s all talk about vaginas for an hour.’ That doesn’t really happen, even though I’m in an office with women, we talk about lots of things but not that. (Lori)

By prefacing her statement with the comment that the topic of CCS is not widely discussed, Lori demonstrates that she does not feel she or any of her female co-workers would consider raising it and that it is not a viable topic of conversation. Lori notes that social constraints and accepted topics for workplace interactions are not barriers when discussing ‘lots of things’, but that CCS is not an appropriate incursion into typical female social discourse. Lori’s first sentence strongly demonstrates her stance that CCS is a significant personal event, and only once ‘you go through it’ can you acquire the knowledge of its implications.

Amy found the inability to discuss CCS and the resulting lack of knowledge highly frustrating:

We really should be spoken to- I don’t even know what they use, I don’t even know what the equipment is, I don’t know nothing about it, only about the fact that you gotta do it [CCS], and it hurts. (Amy)

The only information Amy reports having acquired about CCS is that it involves equipment, pain and is necessary. These uncomfortable certainties are reflected by the use of ‘should’, suggesting young women are being done a disservice by the ‘holder’ of the knowledge, illustrated by the ‘they’ in the first line of the quote. This subordinate theme represents the little knowledge that participants felt they had but also their feelings of exclusion, limiting their ability to gain more information through open discussion.

## Anticipating a Judgemental Exchange

Some participants had already formed uncomfortable expectations from previous pelvic examinations:

During pelvic examinations that I’ve had in the past, it would have been extremely painful and I would have been like, ‘you need to get your finger out of me now because this is excruciating’ and they’ve been kind of impatient and like, ‘we need to do this, come on’, then that negative feedback for me has been like I’m being an inconvenience. (Blaire)

In retelling her previous experience, the immediacy with which Blaire requests the physician stops the examination is countered by the physician’s insistence they continue. The physician frames Blaire’s pain as a necessity for the examination, coupled with the inference they are acting in Blaire’s best interests. Yet ‘get your finger out of me’ conveys Blaire’s strength of reaction and she reflects upon the negative psychological impact of the healthcare professional’s impatience, such that she describes feeling like ‘an inconvenience’. This implies she has felt the exchange derogates her sense of value and crosses her personal limits. Blaire’s experience sets up an expectation that intimate examinations are associated with pain and the removal of autonomy. Hayley’s experience was analogous:

I’ve gone for my endometriosis check-up and I’ve had a bit of strange discharge on the day and they automatically jump to ‘it’s an STI’, then they start doing these swabs and it’s ‘oh well, you know, you’ve clearly done something’, and that’s always a worry for me ‘cause I feel like if I was to go for my cervical cancer screening that it won’t just be that [CCS], that they’ll be like ‘oh well’ and start checking for all these other things [sexually transmitted infections] as well. (Hayley)

Hayley felt that the healthcare staff made assumptions about her sexual (mis)conduct whilst attending a past check-up and this has left her concerned that attending CCS will make her vulnerable to assumptions being made about her body and character again. The pointed suggestion that Hayley could be at fault primed her expectation of future health checks, thereby decreasing her confidence that she would be treated neutrally at CCS. This theme demonstrates how participants’ prior experiences of pain, combined with perceived judgement by healthcare professionals, has negatively primed their expectations of likely processes when attending CCS.

## Solace in One of Many

Participants took comfort in knowing they will not be alone in having CCS. For Vanessa, the fact that many will also be having a CCS, gave comfort: “It makes you not feel singled out that you’ve got to have a test, you’ve got to be examined intimately, it’s comforting to know every woman should experience one in their lifetime” (Vanessa). Vanessa’s response implies discomfort with the intimate nature of the procedure. However, knowing all women should attend CCS diffuses this and provides a rationale for overcoming this reticence. Millie also gained comfort from the doctor’s experience: “You feel very vulnerable in those situations but that’s sort of it really, apart from that they’ve seen it all before, so it doesn’t bother me” (Millie). Vulnerability felt unavoidable but by juxtaposing it with the doctor’s experience Millie was able to neutralise her worries.

Similarly, Lori found being one of many reassuring: “Don’t worry about it, the doctor’s seen 20 vaginas already in the morning, it’s okay, they’re not gonna care” (Lori). Lori references the number of patients a doctor sees, using this to rationalise that the doctor will not be affected by seeing her vagina and she takes solace in being just another number. This theme represents the sense of psychological safety in numbers which was echoed throughout the group, some referencing the doctor’s experience with other group members and some valuing the protective psychological benefits of being lost in the crowd.

### Confronting Sexual Connotations

This superordinate theme demonstrates how CCS and sex were entangled for many participants, although the consequence of this varied greatly.

## The Salience of Sex

Participants did not always consciously make reference to sex when framing CCS: “I would feel very uncomfortable if a male nurse was going down on me, [laughs] not like that” (Katie). Katie’s language use offers an innuendo linking the gender of the health care professional and the procedure with oral sex: her laugh and immediate correction suggests she registered her slip as Freudian. Jo makes a similar concession “You can’t help but think I wonder what his wife’s [vulva] looks like, you can’t not think about that” (Jo). When Jo’s practitioner is male, she almost involuntarily discusses how the gender of the practitioner provokes comparative sexual imagery.

Blaire makes a more direct connection: “When I’m with a male practitioner, it’s like, I’m a female, I have this part, that’s a man, he has a different part. Sex! (laughs)” (Blaire). Blaire speaks of being ‘with’ a male practitioner in the same way she might refer to a partner. However, she then switches to childlike phrasing; the term ‘part’ used to desexualise her presentation. The change in Blaire’s linguistic style suggests she’s revealed discomforting thoughts; she starts to self-censor and by laughing shields the embarrassment over conceding that she thinks of sex. The salience of sex was not dependent on the doctor’s gender; “the speculum does go into the vagina and it’s not obviously sex, but it is that feeling if you know what I mean” (Hayley). Hayley differentiates the procedure from sex but suggests the insertion of apparatus makes the experience physically similar. Ending with ‘if you know what I mean’ suggests Hayley is tentative in her assertion, looking for assurance that she is not alone in making the connection.

For Katie the penetrative nature of CCS invoked a sense of shame: “You’re just letting a stranger in your vagina, like everything you’ve been taught about not letting people do certain things, so it’s like your belief system is questioned” (Katie). Katie omits the stranger’s profession and the purpose of CCS; the emphasis is she would be complicit in allowing herself to be penetrated by a stranger. This theme demonstrates that although the salience of sex during CCS is normally unspoken, it is present.

## Projecting Porn-Like Standards

Many of the participants reported or held themselves to standards seen in porn:

You see their foofs [vaginas] and it’s like looking at a seven-year olds vagine [sic], and you’re like ‘oh no, everyone’s got a different foof’. I’m going to call it a foof because I think it’s a much nicer word. (Camilla)

Camilla reveals an uneasiness with the anatomical term vagina. Opting for the ‘nicer’ term ‘foof’ Camilla creates a softer, more feminine image. Contrasted are the ‘vagines’ which she sees in porn, which are childlike and not representative of real bodies. However, Katie infers people may take on some of these expectations: “if a boy sees a hairy vagina they might run a mile, so why would you feel confident going to a doctor to show them your hairy vagina if you’ve been taught that?” (Katie). Katie uses the idiom ‘run a mile’ to explain that showing a partner a hairy vagina may lead to rejection. Katie does not specify where she learnt this, leaving the impression she sees it as ubiquitous. Katie reasons that having pubic hair would inhibit the confidence needed to attend CCS. Hayley mirrored these concerns:

I do worry that they are going to judge you maybe for like how it looks or you do just panic and you think, my God, is it gonna smell? Have I remembered to shave or have I not shaved? (Hayley).

Hayley refers to her vagina as ‘it’, distancing herself from that part of her body and a potential ‘smell’. Hayley’s fear of judgement reinforces there is a normal expectation of looks, hair and smell, which should be matched and to deviate from this standard, therefore inviting judgement, is panic inducing. This theme highlights the sense of a ubiquitous expectation that vaginas needed to be maintained and hairless, either for the direct benefit of others, to negate potential judgement or to bolster one’s own confidence when considering CCS.

## The Comfort of Female Familiarity

All but one participant expressed a preference for a female practitioner. In previous examinations Jo has felt more at ease: “when it’s a woman doing it, it’s less invasive, it’s a lot more relaxed, you can kind of have a joke with them”. The inclusion of humour during CCS suggests a level of comfort which is fostered with a female practitioner but may not be appropriate with a male. This humour brings Jo and the practitioner psychologically closer; the procedure feels less (psychologically or physically) invasive as they have shared in an experience. Lori’s preference was more considered: “A female gynaecologist would have a lot more understanding purely because she has the same body parts as me; she knows what it feels like to have it (CCS)”. For Lori, a female practitioner has superior empathy and knowledge as she has an implicit and unexplainable physical understanding of the CCS procedure. In sharing the same body parts, Lori predicts a female practitioner will understand her experience better, inferring she will receive better treatment. Millie widens the context of female-to-female understanding, talking about male-to-female sexual harassment and conduct:

If I, as a person that is attracted to women, can go round on my day-to-day basis not smacking people’s bums and shouting at things, then why can’t they [men]? Because it’s exactly the same. I’m attracted to exactly the same type of people as they are, but I don’t do that, but they do, because I understand what it feels like to be on receiving end and they don’t. (Millie)

Referencing how men might interact with women through sexualised interactions, Millie argues that because as men have not lived a female life, they cannot understand how that feels — only female-to-female exchanges allow that shared knowledge. This theme highlights that working with female practitioners would feel familiar enough to participants that they could trust her with jokes, with their bodies, and with the knowledge she would have some understanding of feeling sexually vulnerable too.

### Growing pains

Present in every interview was a sense that this generation faces issues uncharted by the generations before.

## Contending with Comparison Culture

Every participant made reference to damaging comparisons made possible through social media.

20 years ago, you’d be going to school, going out then you’ll come home and that was that and it was fine, but now you can’t escape it and there’s always someone better looking than you on Instagram. Within one click there’s someone better, millions of people better looking than you, and it just doesn’t make you feel good about yourself. (Katie)

Making comparisons with the past, Katie presumes that before social media things were ‘fine’, implying the present culture is no longer ‘fine’. She describes the ease by which social media invokes self-comparison, suggesting it is something worth avoiding. Katie speaks of the role of Instagram in encouraging comparison and its negative impact on her self-concept. Millie also found the comparisons inescapable: “I think that the desirable body image is one thing and I don’t necessarily fit into that and because it’s reinforced constantly in every aspect of our lives it’s very difficult to not compare yourself”. Millie’s indication that it is ‘difficult not to’ make comparisons suggests it is something she would like to abstain from. However, without a safe space from the ‘desirable body image’, Millie’s capacity for comparison abstinence has become depleted. Hayley also saw this as a problematic:

Platforms like Instagram have perpetuated this comparison culture where you just see these things that aren’t real on these platforms like porn and Instagram, and we will compare ourselves to these things because we get told they’re reality and how we should be but when actually they’re not and it can be really damaging. (Hayley)

Hayley spoke of the mechanisms intrinsic to social media as triggers which sustain a culture of comparison. Although she recognises that what is presented is likely detrimentally false, she discloses how it provokes active self-comparison, seen in her use of the phrase ‘we will compare ourselves’. Katie argued that this comparison extended to CCS: “Society and the media have portrayed my body image so negatively, it just doesn’t make me want to show it to anyone, even for health benefits” (Katie). Katie’s perception of her body holds significance in health-related choices. By attributing the source of her discomfort to ‘society and the media’ she demonstrates the power these external influences have on her internal state, depleting her willingness to undertake health screening. This theme demonstrates the effects of becoming intertwined with an unrelenting culture of comparisons via growing up in the digital age.

## Becoming the Focus

In response to ubiquitous beauty standards and a comparison culture, participants had come to focus on themselves:

To some people it sounds big-headed to say you look in the mirror. I look at myself in the mirror and I think ‘oh, I actually look really pretty today, I look really good’. It took A LOT to get to that point. (Sarah)

Sarah understands that self-appreciation can be considered vain but discloses she does not conform. Emphasising that liking her appearance came after considerable effort, she reveals a purposeful dismissal of self-comparison and self-deprecation in pursuit of feeling good. For others, it was a partner’s acceptance which altered their thinking: “The boyfriend that I’m seeing now is fantastic; he makes me feel so secure and he’s very lovely that I think I’ve kind of just lost that anxiety about when I go.” (Hayley). Through being with a caring partner, Hayley has been able to shed insecurities culminating in decreased anxiety over attending CCS. Camilla’s change in focus seemed more related to maturation: “as I’ve got older, I’ve just thought I don’t really care what other people think” (Camilla). Acknowledging a shift in her perspective, Camilla has come to a realisation that other people’s thoughts are inconsequential to her. This facilitates a self-focus which increases willingness to engage with screening: “I think if you’re more concerned about the sexual side of things then you’re not really putting yourself first” (Vanessa). Vanessa advocates for herself by rejecting concerns over sex. For Vanessa, health is a priority and therefore CCS is a duty of self-care. Collectively, this theme demonstrates the ways in which the participants are actively or inadvertently shifting from a focus on others to becoming more assured in themselves, increasing their likelihood of CCS uptake by proxy.

## Discussion

The aim of this study was to explore how awareness, body image and sexual connotations of CCS influence the likelihood of young women attending their first CCS. Interpretative phenomenological analysis identified three superordinate themes: (1) building screening expectations, (2) confronting sexual connotations and (3) growing pains. Together, these themes demonstrate the variance in the amount and quality of available information before attending screening, the entanglement of sex with CCS, and the challenging journey which must be navigated to reject comparison culture.

The present study demonstrated low awareness of CCS knowledge, but also a lack of open dialogue, supporting the notion that CCS is taboo.^22,23^ Yet, having adequate knowledge helps individuals feel prepared for CCS.^11^ A recent systematic review reported a myriad of health education methods based on behaviour change theory which are effective in modifying CCS knowledge and uptake behaviour: tailored counselling, group discussions, self-learning, lectures, postcards, phone calls, radio broadcasts, PowerPoint slides, brochures and mother/daughter education.^42^ Whilst this review demonstrated the utility of theory-based interventions, the current study demonstrates that there is a persistent lack of knowledge in younger people approaching CCS, which needs to be rectified.

Those who had prior pelvic examinations had experienced physical and psychological discomfort, which is highly pertinent; although future CCS uptake is predicted by previous CCS uptake,^42^ negative exam experiences can decrease likelihood of later attendance.^4^ However, those without their own experiences to draw on found comfort in group membership, framing CCS as not a personal experience but an experience shared by a psychological group. Self-categorisation theory^43^ posits that when the self is categorised as belonging to an in-group, the self is no longer considered an individual but a prototype of the in-group via a process of depersonalisation. In the CCS context, the social category of *female* becomes salient, with group members becoming exemplars of a female in-group who collectively take on a group norm of attending CCS. In the current study, categorising the self as part of this psychological group appeared to facilitate CCS attendance intentions. Interventions may benefit from further priming this to increase CCS uptake.

The current study demonstrated that the strong sexual connotations of CCS complicate attendance. Supporting previous findings, participants displayed embarrassment^17,22,23^ and noted concerns over partner opinions,^3,4^ being penetrated,^23^ triggering traumatic memories,^44^ and their vagina’s appearance.^17^ Future campaigns may benefit from acknowledging sexual connotation of the CCS procedure as the elevated prevalence of sexual salience within this group suggests it is particularly powerful for young women. Participant preferences for female practitioners were clear, reflecting perception that female practitioners minimised feelings of sexualisation within the procedure and enhanced female-to-female understanding. This is in accordance with current literature demonstrating consistent preferences for female healthcare professionals in CCS.^3,5,24^ This may reflect the fact that female physicians are experienced as more empathic than their male counterparts.^45–47^ Empathy, in combination with shared experience may intensify the preference for a female physician for an intimate exam. Attendance may be increased by reiterating the option of having a female practitioner.

The present study qualitatively supports previous literature referencing porn and Instagram as detractors of self and body-confidence.^14–16^ This can be understood via Objectification theory^48^ which postulates that women and girls become aware of their sexual objectification and inadvertently take on an externalised view of themselves. Placing heightened worth in their body results in their seeing themselves as an object to be evaluated. Social comparison theory^49^ argues that individuals evaluate themselves by comparison with others. Upward comparisons with individuals viewed as superior can decrease self-regard. Furthermore, when evaluating oneself negatively against perceived cultural ideals, an individual may feel shame about one particular quality, but apply it to themselves in totality.^50^ The present study revealed that social media and CCS both engendered a culture of comparisons, resulting in negative emotions felt towards one’s body, which acted as a barrier to CCS uptake willingness.

**Figure 2:**
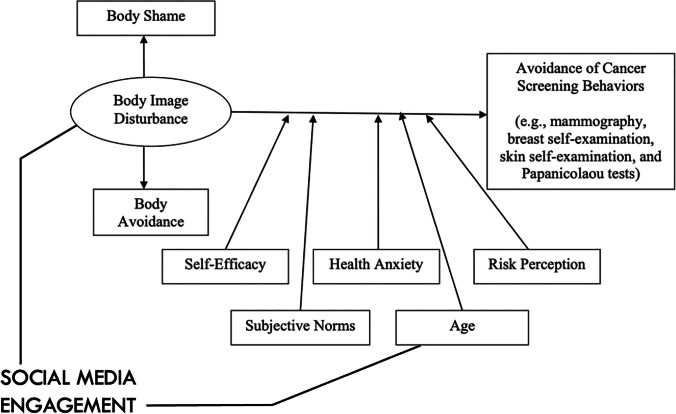
Ridolfi and Crowther’s (2013) model with the inclusion of social media engagement

The current study also demonstrated that this negative relationship with one’s own body was particularly salient for participants. Ridolfi and Crowther’s (2013) model posits a relationship between body image disturbance and body-focused cancer screening among females. The model notes body image disturbance manifests in body shame and body avoidance. Both are demonstrated in the current study as contributing to CCS uptake likelihood. The model includes concepts from health behaviour theories salient for cancer screening; self-efficacy; health anxiety; risk perception; and subjective norms. The only demographic variable in the model is age, included as cancer risk increases with age. However, now that people who grew up with social media are approaching cancer (and CCS) screening age, its inclusion seems even more pertinent: age, via social media and comparison culture exposure, may be a contributing factor to body disturbance, as shown in Fig. 2. This further bolsters the need for tailored body-centred interventions to target young women who are yet to attend CCS.

The articulation and identification of objectification and how it is harmful have been suggested as a method of preventing women from internalising objectification.^51^ Low self-objectification is indirectly linked to preventative sexual health behaviours via body appreciation.^52^ As CCS has sexual connotations it is possible the same mechanisms may function: preventing internalised objectification via articulation/identification may decrease self-objectification and increase CCS attendance via body appreciation. Eighteen to twenty-five-year-old women have been found to be particularly low in body appreciation, decreasing their willingness to discuss sexual issues.^53^ It may be inferred that low body appreciation is therefore putting young women at heightened sexual risk, and risk of rejecting screening. Low body appreciation may be increasing cervical cancer risk whilst decreasing likelihood of CCS uptake. Further research is needed to establish this potential link.

In the current study self-objectification was not stable, as participants reflected on a move towards self-compassion. Self-compassion has been positively linked with health promoting behaviours.^54–56^ Additionally, the body positive movement online is tentatively being forwarded as beneficial for individuals’ body confidence^57^ and increased exposure to ‘real’ images online has been shown to decrease body dissatisfaction.^58^ This may increase self-compassion and therefore health promoting behaviours such as CCS. Self-compassion may therefore be a viable target for CCS uptake interventions in women approaching screening.

## Limitations and Future Research

As per the hermeneutic circle, this study is the interpretation of the researchers and other possible interpretations may be valid.^40^ The small, qualitative and in-depth nature of the study has both strengths and weaknesses, but it provides valuable experiential context informing the rationale behind the uptake decisions planned by young women who represent the first cohort receiving the HPV vaccine. It is suggested future research quantitatively addresses the salience of sex in CCS and the impact of body image in this population. This study looked at intentions of people who are yet to attend CCS, but this was not validated through longitudinal attendance tracking. Future longitudinal studies exploring whether sexual salience, body image and their implications decrease after CCS attendance would be beneficial to understand whether these issues persist. The policy implications of this research are significant in terms of how both mandated and/or optional preventative vaccination and screening are experienced and evaluated. As policies vary widely due to differences in political systems and healthcare services, future research could aim to clarify the extent to which region-specific practices, policies and educational initiatives affect the lived experiences of women approaching their first CCS appointment.

## Implications for Behavioural Health

Attending cervical cancer screening is a protective health behaviour. The current study demonstrated the presence of several barriers to engaging with CCS, namely a lack of CCS knowledge, sexual connotations of CCS and growing up in a culture of comparison. This study lends support to Ridolfi and Crowther’s (2013) model and suggests the necessary addition of social media engagement, as a contributing factor in the relationship between body image disturbances and body-focused screening behaviours among people of female sex. Taking a holistic view to cancer screening behaviours, alongside considerations such as location and time, will enable practitioners to build more effective interventions. Furthermore, the impact of social media engagement may not be solely present in younger women and investigations into other age groups and forms of screening are required.

The present study indicates that women who are yet to attend CCS have little awareness of the procedure, its processes or implications. Interventions using health education methods with theoretical backing are suggested to increase awareness,^41^ but still need to target young people who have not yet received a CCS invitation. CCS has sexual connotations which contribute to embarrassment,^17,22,23^ concerns over partner opinions^3,4^ and may trigger traumatic memories.^44^ The findings of the current study extend and bolster the case for a move towards self-sampling at home,^3,59^ which may circumvent these potential barriers, alongside traditional barriers such as time and location.

## Data Availability

The data that support the findings of this study are available from the corresponding author upon reasonable request.

## Footnotes

1 Given the complexity of gendered terms in health research, the researchers wanted to acknowledge the choice to use the terms ‘women’ and ‘female’ throughout the article. This was chosen as the research and psychological model the study references refer to women, and as participants in the study were female identifying.
